# Parapharyngeal Space Ganglioneuroma in a Child: A Report of a Rare Case

**DOI:** 10.7759/cureus.89493

**Published:** 2025-08-06

**Authors:** Canh T Pham, Ha T Nguyen, Tuan A Le, Thinh D Dang

**Affiliations:** 1 Otolaryngology, Hanoi Medical University, Hanoi, VNM; 2 Head and Neck Surgery, National Otolaryngology Hospital, Hanoi, VNM; 3 Pediatric Otolaryngology, National Otolaryngology Hospital, Hanoi, VNM; 4 Pathology, Hanoi Medical University, Hanoi, VNM

**Keywords:** ganglioneuroma, parapharyngeal space, pediatric neck mass, transcervical approach, tumor

## Abstract

Parapharyngeal space (PPS) tumors are extremely rare in the pediatric population, accounting for a small fraction of all head and neck neoplasms. The majority of neoplasms in the PPS are benign tumors. We present a case of an eight-year-old male with a large PPS ganglioneuroma, who presented with a neck mass that had been progressing over five years with no symptoms of dysphasia. Diagnostic imaging, including computed tomography scan and magnetic resonance imaging with contrast, revealed a large, heterogeneously enhancing mass, measuring about 4.47 × 6.49 × 2.56 cm in size, in the right poststyloid PPS. The tumor extended closely from the common carotid artery bifurcation to the skull base, without intracranial extension. A transcervical surgical approach enabled complete tumor excision without complications. Postoperative histopathology confirmed the diagnosis of ganglioneuroma, a very rare case in pediatric PPS tumors. No postoperative complications were noted, and follow-up over two years revealed no signs of recurrence.

## Introduction

Tumors of the parapharyngeal space (PPS) are exceedingly rare, accounting for only 0.5% of all head and neck neoplasms. The PPS is an anatomical region that has the form of an inverted pyramid comprising a base, a vertex, and three walls. The PPS is a well-defined fascia space bounded superiorly by the skull base and inferiorly by the hyoid bone and enclosed medially by the buccopharyngeal fascia along the craniocaudal axis. It is bordered by the retropharyngeal space posteromedially and the carotid sheath posterolaterally. The fascia running posteriorly through the styloid process to the tensor veli palatini muscle divides the PPS into the prestyloid and poststyloid compartments. Due to their deep location within a potential space, PPS tumors may remain asymptomatic for extended periods, allowing them to reach considerable size before clinical detection. The majority of PPS tumors, approximately 80%, are benign in nature, while malignant lesions represent only about 20% [1,2]. The most common tumor types are of neurogenic (35-41%) or salivary (35-45%) origin, whereas other histological types, such as meningiomas, hemangiomas, or lipomas, are tremendously rare [3]. The primary imaging modalities currently used for evaluating PPS tumors are contrast-enhanced computed tomography (CT) and magnetic resonance imaging (MRI) [4]. Most benign PPS tumors can be surgically excised with a low rate of complications and recurrence. Among the various surgical techniques, the transcervical approach is the most commonly employed [5]. Because of the complex anatomy of deep location and critical structures within the region, surgical resection of PPS tumors can be technically challenging and associated with specific risks, particularly when the tumor extends toward the skull base or far medially. Herein, we report a rare case of ganglioneuroma of the PPS in an eight-year-old male child with a five-year history of a painless neck mass. The tumor was successfully excised via a transcervical approach, with histopathology confirming ganglioneuroma. No recurrence was observed during the two-year follow-up period. This appears to be the first documented case of a parapharyngeal ganglioneuroma in a Vietnamese child, and such cases remain exceedingly rare worldwide.

## Case presentation

**Figure 1:**
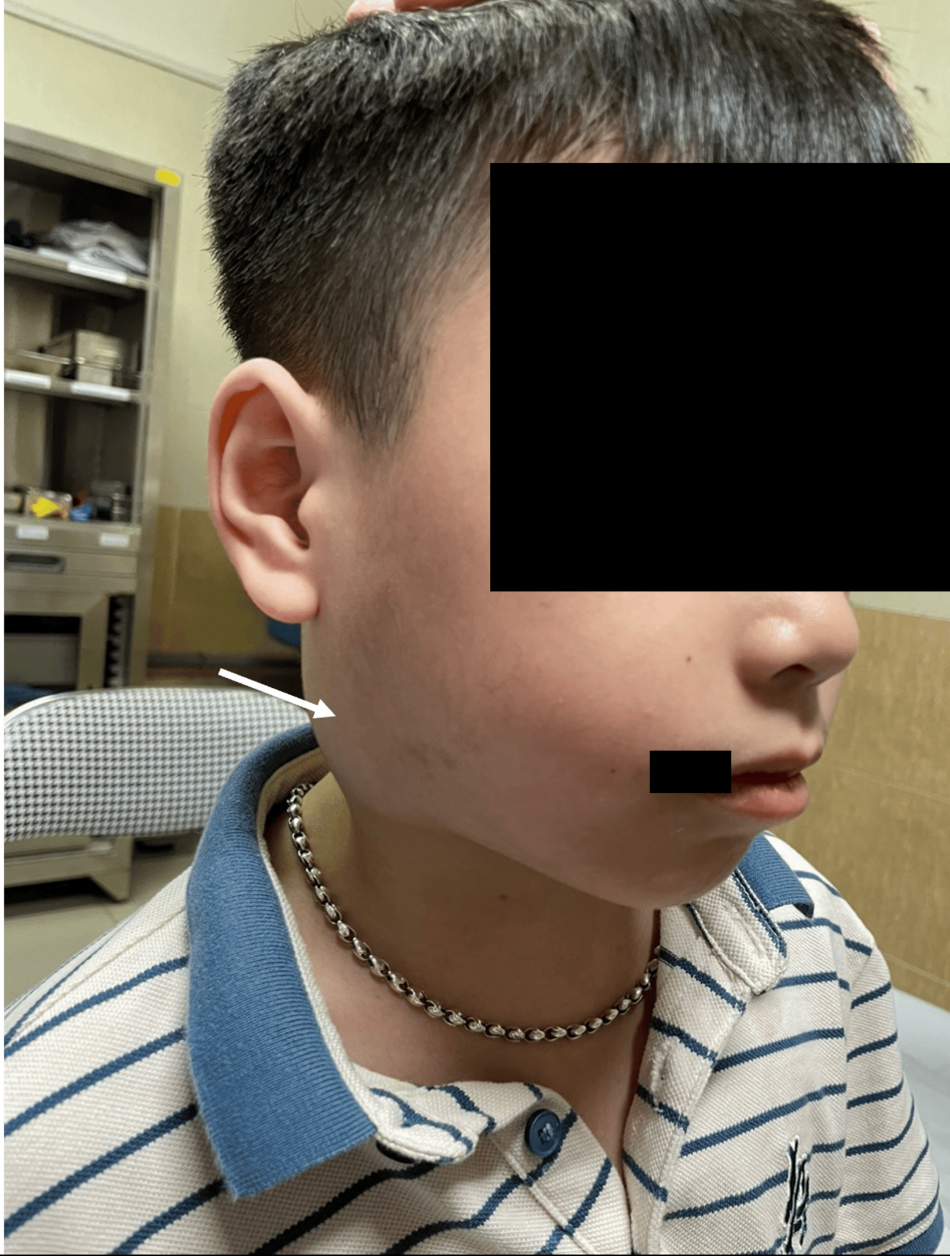
Preoperative clinical assessment image of an eight-year-old male child with a mass (white arrow) on the right side of the neck.

An eight-year‑old male child presented to a surgeon with a five-year history of progressively enlarging, painless lump on the right side of the neck with no history of weight loss, dysphagia, or difficulty with mastication (Figure 1). There were no symptoms of dysphonia, dyspnea, or obstructive sleep apnea observed in the patient.

Prior treatment with anti-inflammatory and antibiotic medications at a local clinic had been ineffective. There was no history of allergies or other medical conditions. The child had undergone tonsillectomy and adenoidectomy one year prior for recurrent tonsillitis, performed by another surgical team.

**Figure 2:**
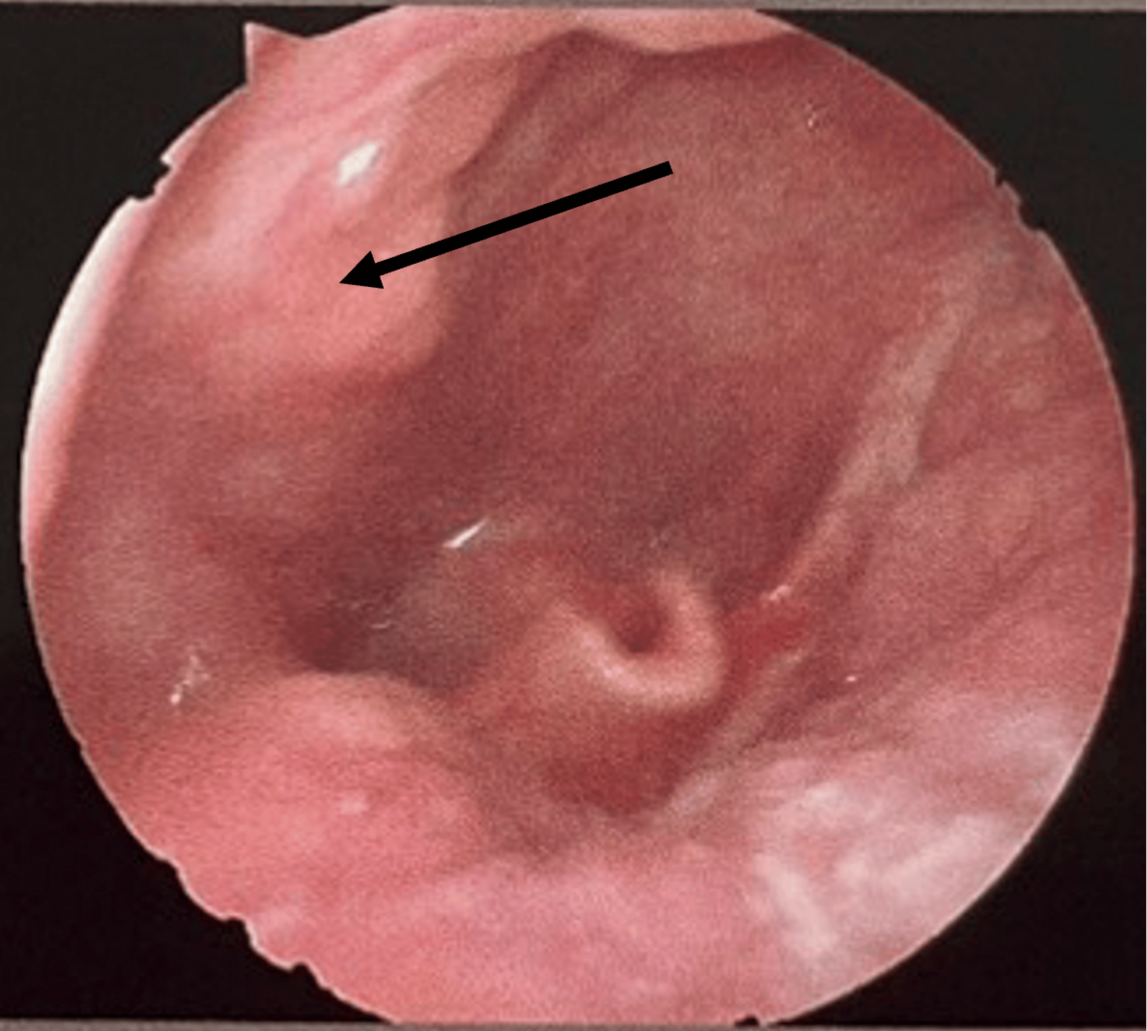
Endoscopy examination: tonsils were removed, and a swelling on the right side wall of the oral pharynx was noted (black arrow).

On physical examination, there was a diffuse mass on the right side submandibular region of the neck, measuring approximately 7.0 × 4.0 cm. On palpation, the lesion was non-tender, non-fluctuant, smooth-surfaced, partially mobile mass, with normal overlying skin and no cervical lymphadenopathy. Oropharyngeal examination revealed a smooth bulge on the right side wall of the oral pharynx. The tonsils had been removed in the previous surgery (Figure 2). The remainder of the ENT examination was unremarkable.

**Figure 3:**
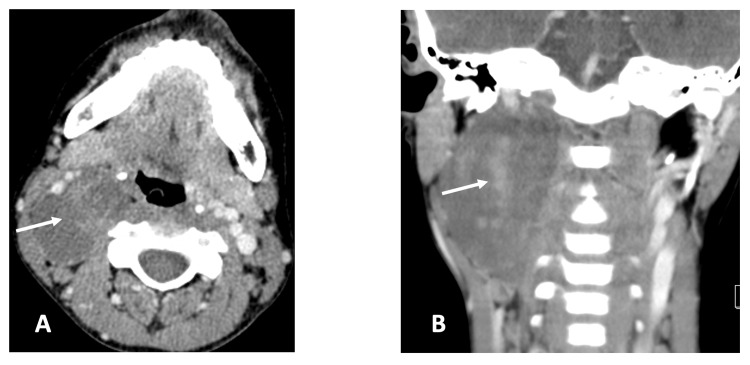
Axial (A) and coronal (B) views of contrast-enhanced CT images demonstrating a mass (white arrow) occupying the right parapharyngeal space.

A contrast-enhanced CT scan revealed a heterogeneously enhancing mass measuring approximately 4.47 × 6.49 × 2.56 cm, located in the right posterior PPS and posterior cervical space, displacing carotid arteries and jugular vein anterolaterally (Figure 3).

**Figure 4:**
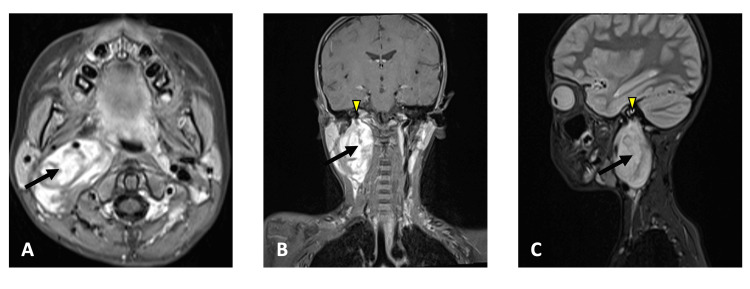
Axial (A), coronal (B), and sagittal (C) contrast-enhanced MRI demonstrating a tumor (black arrow) within the parapharyngeal space extending superiorly toward the skull base (arrowhead).

MRI corroborated the CT scan findings, demonstrating an iso-intense lesion on both T1- and T2-weighted sequences with heterogeneous enhancement following contrast administration (Figure 4). The mass extended from the level of the common carotid artery bifurcation to the skull base, without evidence of intracranial extension. Imaging features on CT and MRI were suggestive of a benign neoplasm.

Based on the clinical presentation and imaging findings, a tumor was identified in the PPS. In pediatric patients, the differential diagnosis typically includes benign tumors such as pleomorphic adenoma of the salivary glands, schwannoma, neurofibroma, and ganglioneuroma.

**Figure 5:**
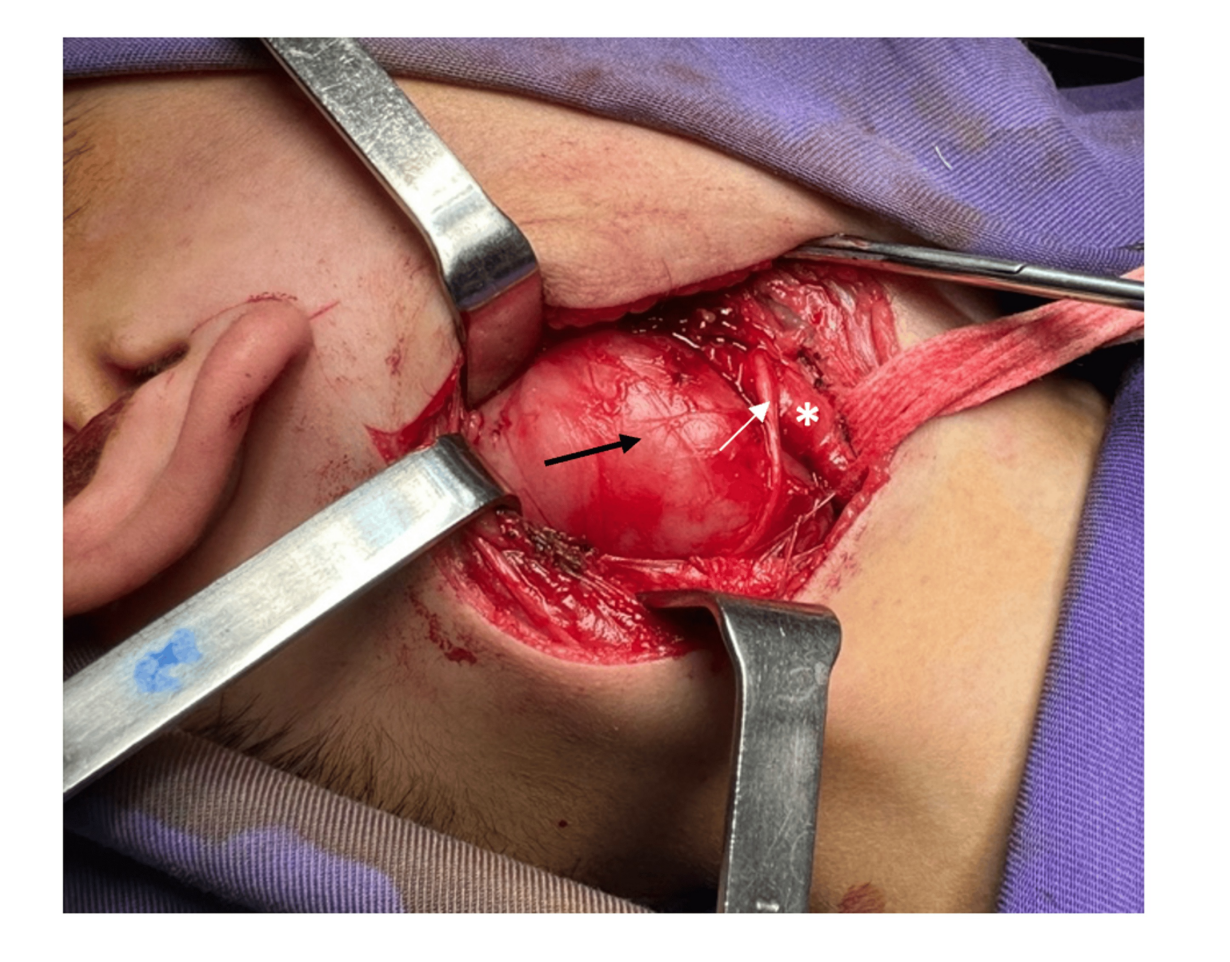
Intraoperative view following elevation of the subplatysmal flap shows parapharyngeal space tumor (black arrow), ansa cervicalis (white arrow), and common carotid artery bifurcation (asterisk).

**Figure 6:**
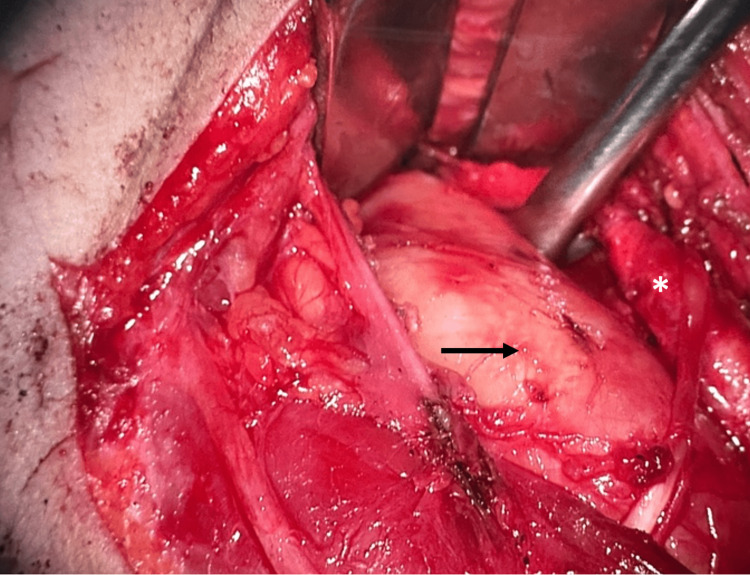
Intraoperative dissection demonstrating the separation of the tumor (black arrow) and internal carotid artery (asterisk).

Tumor resection of the PPS was performed via a right-sided transcervical approach with vertical skin incision. Intraoperatively, the tumor’s location and its relationship with adjacent vascular structures were carefully assessed (Figure 5). Precise dissection was conducted using a microscope, with particular attention to the carotid triangle, allowing for safe separation of the mass from the internal carotid artery and internal jugular vein (Figure 6). Following complete excision of the tumor, meticulous hemostasis was achieved, and preservation of the vagus nerve and facial nerve branches was confirmed prior to wound closure. The postoperative course was uneventful, with no complications observed.

**Figure 7:**
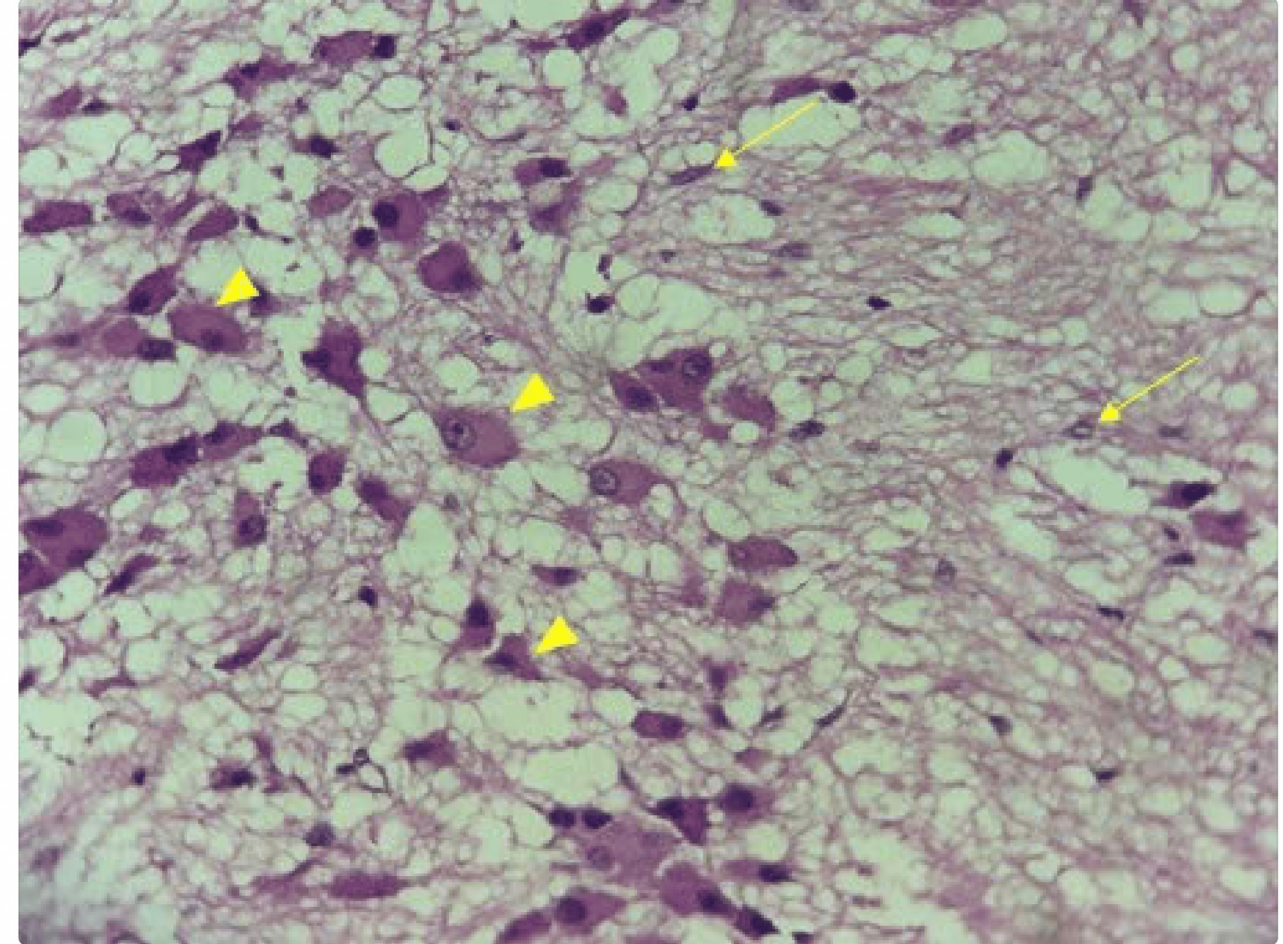
Microscopic image of the ganglioneuroma tissue (hematoxylin and eosin, x400) consisting of Schwann cell components (arrows) and ganglion cell components (arrowheads) proliferating on a background of myxoid stroma.

Gross examination revealed a well-encapsulated, circumscribed, soft mass in consistency, measuring approximately 7.0 × 4.0 × 3.0 cm. Histopathological analysis demonstrated regions composed of multipolar cells with abundant cytoplasm and prominent nuclei, interspersed with areas of diffuse hyperplastic Schwann cells embedded in a fibrillar and myxoid stroma (Figure 7). The final postoperative diagnosis was ganglioneuroma of the right PPS.

## Discussion

The PPS, situated within the suprahyoid region of the neck, is a complex anatomical area resembling an inverted pyramid. It is divided into two distinct compartments: the prestyloid (anterior) and poststyloid (posterior) regions. The anterior (prestyloid) compartment predominantly contains minor or ectopic salivary glands, branches of the mandibular nerve, the internal maxillary artery, the ascending pharyngeal artery, and the pharyngeal venous plexus. Conversely, the posterior (poststyloid) compartment encompasses vital neurovascular structures, including the internal carotid artery, internal jugular vein, cranial nerves IX, X, XI, and XII, the cervical sympathetic chain, and lymph nodes.

Tumors of the PPS are uncommon and exhibit a broad range of histopathological subtypes, with approximately 80% classified as benign [1,2]. Among the various neoplasms that can originate in this region, salivary gland and neurogenic tumors are the most prevalent [6]. Tumors arising in the prestyloid compartment are predominantly of salivary gland origin, whereas those in the poststyloid compartment are primarily neurogenic in nature [7,8]. The average age at presentation is approximately 46 years, reflecting a heterogeneous spectrum of histological diagnoses [2,7]. PPS tumors are exceedingly rare in the pediatric population, posing significant challenges for early diagnosis and surgical management. In a study of 23 primary PPS tumors, only three cases involved patients under the age of 18 years, and none were identified as neurogenic in origin [3,9]. Compared to adults, pediatric PPS tumors exhibit distinct histopathological patterns. While salivary gland neoplasms and paragangliomas are predominant in adults, these entities are exceedingly rare in children, where neurogenic tumors represent the most frequently encountered benign lesions (Table 1) [2,9].

**Table 1:** Comparison of benign and malignant parapharyngeal space (PPS) tumors.

Feature	Benign tumors	Malignant tumors
Prevalence	~80% of PPS tumors	~20% of PPS tumors
Common types	Pleomorphic adenoma, schwannoma, neurofibroma, ganglioneuroma	Mucoepidermoid carcinoma, adenoid cystic carcinoma, paraganglioma, sarcoma
Growth	Slow, longstanding	Rapid, progressive
Symptoms	Painless neck mass, mild dysphagia	Pain, cranial nerve deficits, dysphagia, trismus
Imaging (CT/MRI)	Well-defined, non-invasive	Poorly marginated, invasive
Histopathology	Encapsulated, uniform cells, no atypia	Cellular atypia, mitoses, infiltrative growth
Treatment	Surgery	Surgery ± radiotherapy/chemotherapy
Prognosis	Good with low risk of recurrence	Variable, depending on histological type and disease stage

Ganglioneuroma is a rare, benign, and noninvasive neoplasm originating from the sympathetic nervous system. It is composed of well-differentiated ganglion cells, nerve sheath cells, and nerve fibers. While these tumors most frequently arise in the posterior mediastinum, adrenal gland, and retroperitoneal regions, their manifestation in the head and neck is relatively uncommon. The involvement of ganglioneuroma in the PPS is extremely rare. As a benign peripheral nerve tumor, ganglioneuroma generally demonstrates slow growth. The onset of clinical symptoms primarily depends on the tumor’s size and anatomical location. The PPS, extending from the base of the skull to the level of the hyoid bone, is a deep and relatively accommodating space. Consequently, benign tumors in this area often remain asymptomatic for a prolonged duration, resulting in a delayed clinical diagnosis. The slow, asymptomatic growth over five years in our patient is characteristic of this benign behavior. In a small number of patients, large tumors may exert compressive effects on adjacent peripheral nerves and surrounding tissues, leading to neurological manifestations such as dysphagia, hoarseness, post-deglutition choking, and, in some cases, Horner’s syndrome [10]. Moreover, as the tumor enlarges, it presents a significant challenge for surgical removal and increases the risk of postoperative complications. With low levels of neuroendocrine activity, certain ganglioneuromas may exhibit functional characteristics, leading to symptoms of high blood pressure, sweating, and diarrhea. In our study, the child suffered from a symptom of a neck mass for five years, with no complaint of swallowing obstruction, dyspnea, or other neuroendocrine symptoms.

MRI and contrast-enhanced CT play a central role in the diagnosis and preoperative planning of PPS tumors [4,11]. In this case, a contrast-enhanced CT scan was performed to evaluate the size, location, and relationship of the mass to adjacent vascular and bony structures. MRI was prioritized for its superior soft tissue contrast, allowing precise delineation of the tumor's extent and anatomical relationships within the PPS. Notably, MRI is particularly effective in assessing the tumor’s proximity to critical vascular structures, such as the internal carotid artery, and in characterizing specific neoplasms, including pleomorphic adenomas and paragangliomas. While cervical ultrasound is a non-invasive and cost-effective modality for evaluating superficial neck lesions, its utility is significantly limited in deep anatomical regions like the PPS due to acoustic shadowing and restricted field of view. Fine-needle aspiration can be helpful in differentiating benign from malignant lesions in PPS tumors; however, its application in pediatric patients is often constrained by the tumor’s deep location and the technical difficulty of obtaining adequate samples. Given the imaging characteristics suggestive of a benign lesion and the limitations of fine-needle aspiration in this context, we proceeded directly with surgical excision for both diagnostic confirmation and definitive management.

Surgery remains the cornerstone of treatment for PPS tumors. The PPS presents significant surgical challenges due to its deep anatomical location and the presence of critical neurovascular structures. These complexities necessitate careful operative planning to achieve complete tumor removal while minimizing trauma to surrounding tissues and preserving functional and cosmetic outcomes. Various surgical approaches have been developed to address these demands, including transoral, transcervical, transparotid, and transmandibular techniques, used individually or in combination, depending on the tumor's size, anatomical position, and histological characteristics. Among these, the transcervical approach is most commonly employed due to its favorable access and versatility [6,12,13]. The selection of the optimal surgical approach should be tailored to each case, taking into account available resources, patient-specific factors, and the surgeon’s expertise [12]. In our case, a transcervical approach was employed to access the tumor located in the poststyloid region. This approach enabled precise dissection and control of the lesion, which was limited in its extension toward the skull base and situated in close proximity to the internal jugular vein and the common carotid artery. Given the intricate anatomy of the PPS, potential surgical risks included injury to major blood vessels, the vagus nerve, and the skull base. The most frequent complication after surgery in the literature review is the injury of variable degree to the cranial nerves in the PPS (i.e., cranial nerves VII, IX, X, and XII). There was a greater risk of cranial nerve lesion in cases of neurogenic and malignant tumors. According to the literature, vagus nerve injury represents the most frequently reported complication. The incidence of permanent palsy involving the vagus nerves and the facial nerve is approximately 10.4% and 12.5%, respectively [14]. Additionally, there was a risk of creating an unintended communication between the surgical cavity and the oropharynx, which could lead to postoperative complications. In our case, the tumor was adherent with a lot of vascularity, especially to the sheath of the external carotid artery and the accessory nerve. The tumor was dissected carefully with the protection of all important vessels and nerves.

PPS ganglioneuromas are extremely rare, particularly in children, with only a few cases reported globally. In a previous study of PPS ganglioneuromas, both reported cases involved adult patients [15]. In our case, the patient presented with a PPS tumor located in the poststyloid region, with the first symptoms manifesting at the early age of three years. In another study, one case involved a 2.5-year-old girl who presented with severe symptoms, including obstructive sleep apnea, odynophagia, and stridor. She underwent tracheostomy for airway control, followed by a biopsy and subsequent surgical excision of the tumor. Her postoperative course was complicated by Horner’s syndrome and significant dysphagia with aspiration of thin liquids [16]. Another case involved a two-year-old girl who presented with a progressively enlarging mass located high on the right side of the neck, noted over a two-month period. Intraoral examination revealed an anteromedial displacement of the right posterior pharyngeal wall and no signs of cranial nerve impairment [9]. A third case in children involved a 17-year-old boy presenting with throat pain, dysphagia, and a right-sided oropharyngeal mass. Although the clinical presentation varies among reported cases, a common feature is the presence of a neck mass [17]. In all instances, CT and MRI were instrumental in evaluating the lesion and suggesting a benign etiology. Two of the patients underwent biopsy prior to definitive tumor excision, which confirmed the diagnosis of ganglioneuroma. However, in our opinion, if both clinical and imaging findings strongly support a benign lesion, primary surgical excision without prior biopsy may be appropriate in pediatric patients to avoid subjecting them to multiple invasive procedures. In uncertain cases, image-guided, typically CT-guided, biopsy may be considered prior to surgery [4].

The prognosis for ganglioneuroma is generally favorable, with a low risk of recurrence when complete surgical excision is achieved. The primary concern during surgical management lies in the potential sacrifice of neural structures closely associated with the tumor, which may result in postoperative neurological deficits. In our case, the patient remained stable following surgery, with no evidence of neurological or vascular complications. Notably, there were no signs of autonomic dysfunction, including Horner's syndrome, indicating preservation of the sympathetic chain and surrounding neural elements. A two-year follow-up demonstrated no signs of tumor recurrence in this patient.

## Conclusions

PPS tumors are extremely rare in the pediatric population and present a diagnostic and therapeutic challenge. Diagnosis requires a thorough evaluation of clinical symptoms, detailed physical examination, and appropriate imaging studies, particularly contrast-enhanced CT and MRI. Surgical removal is the first choice in treatment and offers an excellent prognosis. The transcervical route is the most common approach due to its superiority in providing direct entry to the PPS and satisfactory control of neurovascular structures of the neck. However, surgical therapy to remove PPS tumors remains challenging, especially in children, because of the deep position and close relationship to the surrounding vital neurovascular system. Benign tumors that are fully resected typically have a good prognosis with a low risk of recurrence. This case underscores the importance of considering rare neurogenic tumors in the differential diagnosis of persistent pediatric neck masses.
